# PD-L1 Expression and Immune Cell Infiltration in Gastroenteropancreatic (GEP) and Non-GEP Neuroendocrine Neoplasms With High Proliferative Activity

**DOI:** 10.3389/fonc.2019.00343

**Published:** 2019-05-07

**Authors:** Martina Ferrata, Arno Schad, Stefanie Zimmer, Thomas J. Musholt, Katharina Bahr, Julian Kuenzel, Sven Becker, Erik Springer, Wilfried Roth, Matthias M. Weber, Christian Fottner

**Affiliations:** ^1^Department of Endocrinology and Metabolism, I Medical Clinic, University Hospital, Johannes Gutenberg University of Mainz, Mainz, Germany; ^2^Institute of Pathology, University Medical Center of the Johannes Gutenberg University of Mainz, Mainz, Germany; ^3^Clinic of General, Visceral- and Transplantation Surgery, Endocrine Surgery Section, University Medical Center of the Johannes Gutenberg University of Mainz, Mainz, Germany; ^4^Department of Otorhinolaryngology, Head and Neck Surgery, University Medical Center of the Johannes Gutenberg University Mainz, Mainz, Germany

**Keywords:** neuroendocrine carcinoma, immune checkpoint inhibitor, PD-L1, tumor associated immune cell, neuroendocrine tumor, neuroendocrine neoplasm, T cell infiltration

## Abstract

The potential of neuroendocrine neoplasms (NEN) to respond to checkpoint inhibitors is largely unknown and full of great expectations. Immunohistochemical (IHC) studies of programmed cell death ligand 1 (PD-L1) expression in the tumor microenvironment and its implications in predicting the response to checkpoint inhibition is a very active subject. Currently, the combined analysis of PD-L1 expression and tumor-associated immune cell (TAIC) infiltration is considered the best predictive marker of therapeutic response. Here we investigated the expression of PD-L1 on tumor cells (TC) and tumor-infiltrating immune cells (IC) by IHC in 68 NEN samples with a high proliferation rate (Ki-67 >20%) from 57 patients and in 22 samples we correlated it with TAIC density by assessing intratumoral infiltration of CD3+, CD8+, and CD68+ cells. Furthermore, the tumor microenvironment was evaluated according to the classification of Teng et al. We detected PD-L1 expression in 31.6% of NEN G3. Its expression usually was weak and more IC than TC expressed PD-L1. The proportion of tumors positive for PD-L1 was comparable in NEN from different sites of origin but varied depending on tumor differentiation and disease extension. No positive IHC staining was found in 3 well-differentiated neuroendocrine tumors (NETs) with a proliferation rate above 20% (NET G3). When analyzing TAIC, we rarely (18.2%) detected intratumoral CD8+ cells, whereas infiltration by CD3+ and CD68+ cells was more common (45.5 and 59.1%, respectively). By combining CD3+ cells and PD-L1 status, we identified the immune ignorant phenotype of tumor microenvironment as being the most common phenotype, supporting the concept of a preferably combined immunotherapeutic approach in neuroendocrine carcinoma (NEC).

## Introduction

The WHO 2010 classification of neuroendocrine neoplasms (NEN) (1) distinguishes well to moderately differentiated neuroendocrine tumors (NET), which are graded depending on their proliferation status into NET G1 (Ki-67 index < 2%), and G2 (Ki-67 2–20%), from poorly differentiated, highly malignant neuroendocrine carcinomas (NEC G3, Ki-67 >20%). However, NEC G3 is not considered a homogeneous entity. As recently addressed in the WHO 2017 classification of pancreatic NEN (2) a morphologically still differentiated NET G3 subgroup with a Ki-67 in the lower proliferative range usually between 20 and 55% can be separated from the more aggressive and undifferentiated “classical” small or large cell NEC G3 which have a very poor prognosis. The mixed neuroendocrine-non-neuroendocrine neoplasms (MiNEN) represent a usually highly proliferative entity. which is composed of a non-neuroendocrine (most frequently adenocarcinoma) and a neuroendocrine component that, by definition, exceeds 30% of the whole neoplasm. Usually, both components are G3 malignant carcinomas (1).

Although NEC G3 usually respond to cisplatin/etoposide-based chemotherapy, the duration of response is only of short with a progression free survival (PFS) of 4–9 months and an overall survival (OS) of 10–19 months. Grade 3 and 4 toxicity rates are high and there is no standard second line chemotherapy (3, 4). Therefore, the development of novel drugs for the treatment of NECs is crucial. Among them, immune therapy may offer a recent and promising approach (4–6).

The tumor escape mechanisms of how cancer hijacks intrinsic immune mechanisms developed to limit inflammatory and immune responses as well as to protect the host from autoimmunity include loss of tumor antigenicity and/or T cell activation and function, targeting of regulatory T cell function, modification of immune suppressive mediator production, tolerance and immune deviation (7, 8). One important mechanism of tumor immune escape is to block antigen-specific T cell responses by immune checkpoints, which tightly regulate antigen recognition by T cells and their effector functions (9). The binding of cytotoxic T lymphocyte-associated protein 4 (CTLA-4) to co-stimulatory ligands on antigen-presenting cells (APC) prevents T-cell signaling through CD28 (10). On the other hand, the interaction of programmed cell death protein 1 (PD-1) receptors and its ligands PD-L1 (B7-H1, CD274) and PD-L2 (B7-DC, CD273) plays a key role in inducing the adaptive immune resistance (11–14). PD-1 is a transmembrane glycoprotein receptor of the immunoglobulin superfamily with a co-inhibitory function weakly expressed in resting T cells. However, its expression is induced upon activation of CD4+ and CD8+ T cells, natural killer cells, monocytes and B cells. Under physiological conditions, PD-1 activation prevents overstimulation of immune responses in peripheral tissues and thus reduces autoimmunity and promotes immune tolerance. Stimulation of the PD-1 receptor on activated T cells lowers the ability of cells to produce cytokines and hence promotes tolerance to chronically expressed antigens (15–17).

PD-L1 is expressed on tumor and/or immune cells of the tumor microenvironment, i.e., on stromal tumor-associated macrophages and lymphocytes. PD-L2 expression is less intense and primarily restricted to APC. Its functional significance remains unclear and studies investigating tumor-induced adaptive immune resistance via PD-1 checkpoint inhibition mainly focus on PD-L1 expression. In many tumor types PD-L1 expression correlates with response to PD-1/PD-L1 inhibition (18). However, patients with very low or absent PD-L1 expression on tumor cells (TC) still may derive some benefit from treatment (18, 19).

There is growing evidence that PD-L1 expression and response to treatment should best be evaluated in the context of T cell infiltration of the tumor. Teng and colleagues (20) have proposed 4 patterns of tumor microenvironment according to PD-L1 expression and presence of tumor-infiltrating lymphocytes (TILs): adaptive immune resistance (PD-L1 positive/TILs present), immune ignorance (PD-L1 negative/TILs absent), intrinsic induction (PD-L1 positive/TILs absent) and immune tolerance (PD-L1 negative/TILs present). The combined analysis of PD-L1 expression and tumor microenvironment might help to better stratify which patients will benefit from what type of immunotherapy and to prevent a priori exclusion of PD-L1 negative patients who might still benefit from checkpoint inhibitor therapy (21–23). Very little is known concerning PD-L1 status and tumor microenvironment of NEN and so far no data is available on the importance tumor immune phenotypes for the prediction of tumor response to immunotherapy in high-grade NEN (24–26).

Currently, the anti-PD-L1 antibody avelumab is approved for treatment of Merkel cell carcinoma, a highly aggressive neuroendocrine skin tumor. Promising results for checkpoint inhibitors have been found in phase 2 studies for small cell neuroendocrine lung cancer (SCLC) (27–29). In addition, case reports and data from basket studies support the concept of immune therapy in NEN, which is being further evaluated in several ongoing trials [for a review, see (30)]. Studies in other solid tumors have demonstrated that a high mutational burden and neoantigen-rich tumors better respond to checkpoint inhibitors (31, 32). The high aberration rate and typically increased proliferative activity of NEN G3 result in rapid appearance of neoantigens, making the tumors highly immunogenic. Therefore, it seems reasonable to assume that of all neuroendocrine lesions, NEN G3 will be the most appropriate target for successful treatment with checkpoint inhibitors.

Results on PD-L1 expression on NEN are scarce and contradictory, since in most cases only small series were investigated which differed in grading and other major tumor characteristics (33–42). In order to get a more robust report on the role of immunomodulation in NEN we studied the expression of PD-L1 by IHC on TC and tumor-infiltrating immune cells (IC) in a large group of NEN with high proliferative activity (Ki-67 >20%).

## Materials and Methods

### Study Design

The study cohort included 68 formalin-fixed paraffin-embedded (FFPE) tissue samples from 57 patients. PD-L1 staining was performed on each sample. TAIC density requires large tissue samples of complete tumor samples (“sample in toto”); lymph node samples were also excluded to avoid confounding immune cell infiltration; so, small samples and lymph nodes were excluded from this second analysis. All analyses were performed in accordance with the guidelines of the local ethical committee and were based on the written consent of the patients of the tissue tumor bank of the University Medical Center. Only lesions with documented G3 grading according to WHO classification 2010 (1) were included. Samples comprised of primary tumor samples from different sites of origin and/or their metastases. At study inclusion, the pathological diagnosis was reviewed by an expert pathologist in all cases. The histopathologic data collected included: histological tumor differentiation subtype, tumor site of origin, histological staining grade for synaptophysin (Syn), chromogranin A (CgA), and proliferation index (Ki-67). The original pathology reports of the patients were used, all data were collected referring to patients with a code and anonymity was maintained. Tumors were staged according to the actual TNM classification of malignant tumors and were classified according to the latest WHO grading system by site of origin. Finally, disease was staged accordingly into locally confined, locally invasive or metastatic. Due to the fact that surgery is not a therapeutic option in the majority of G3 NEN patients, only initial biopsies had been performed in most of these patients, thus limiting the amount of material available for pathological analysis. For all 11 patients with multiple samples, IHC staining was performed on all samples, but only one sample per patient was included in the final statistical analysis: inclusion criteria were either the PD-L1 positive expression or the sample size. Available medical records, including pathology reports, were reviewed to obtain additional clinical data including gender, age at time of the first diagnosis. In the retrospective setting of our study, it was unfortunately not possible to gather more clinical data, due to the very strict data safety regulations imposed by the local ethical committee.

### IHC Analysis

**Figure 1:**
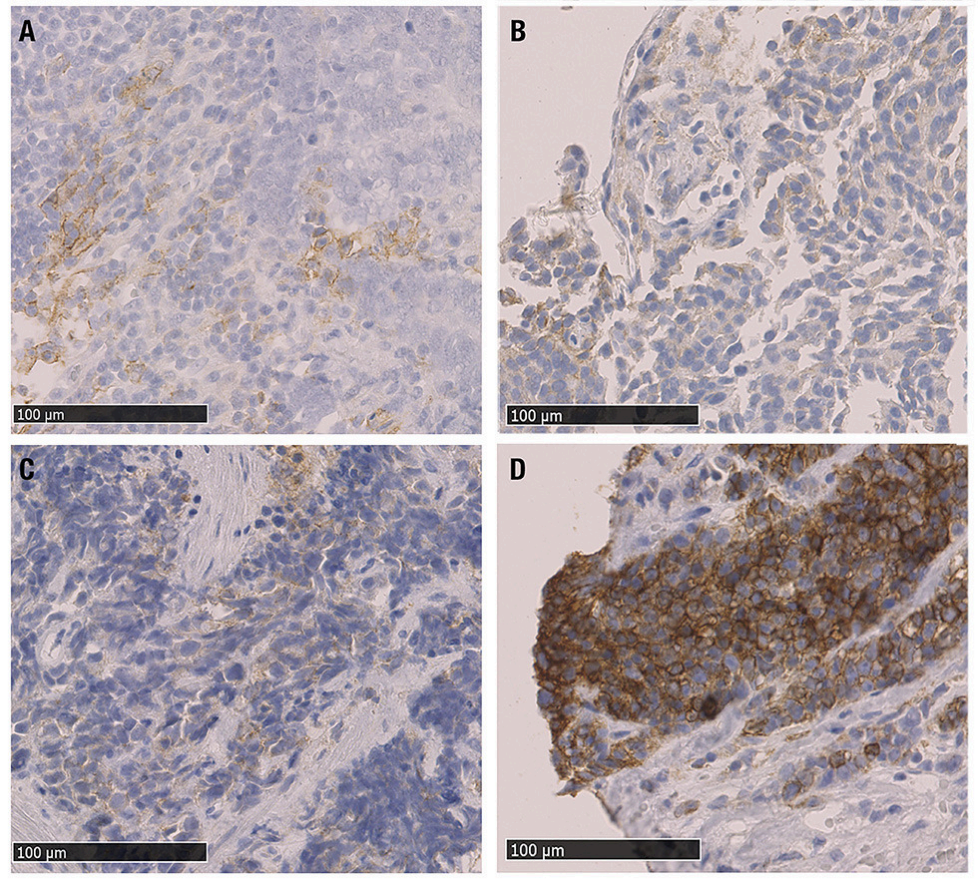
Microphotographs of representative examples of IHC PD-L1 expression in NEN G3 samples; different levels of staining (brown) are shown. **(A)** PD-L1 positive TC (score 1+) (gastrinoma, distant metastasis), **(B)** PD-L1 positive IC (score 1+) and PD-L1 negative TC (Merkel cell carcinoma lymph node metastasis), **(C)** PDL1 positive TC and IC, both score 1+ (lung, primary tumor sample), **(D)** PD-L1 positive TC (score 3+) (colon MiNEN, distant metastasis). PD-L1: programmed cell death ligand 1, IHC: immunohistochemistry, TC: tumor cells, IC: tumor-infiltrating immune cells, MiNEN: mixed neuroendocrine-nonneuroendocrine neoplasm.

Two-micrometer-thick sequential histologic tumor sections were obtained from an archival FFPE representative tumor block and used for IHC analysis. IHC was performed using an automated staining system (Dako Cytomation Autostainer plus, Dako Deutschland GmbH, Hamburg, Germany) with antibodies against PD-L1 (clone EPR19759, dilution 1:250; ab213524, Abcam, Cambridge, UK), CD3 (T cell lymphocytes; FLEX polyclonal rabbit; ready-to-use; Dako), CD8 (cytotoxic T cell; FLEX monoclonal mouse clone KP1; ready-to-use; Dako) and CD68 (macrophages; FLEX monoclonal mouse clone T8/144B; ready-to-use; Dako). Expression of all cellular markers was detected using a detection kit (Dako EnVision™ FLEX detection system) with a diaminobenzidine reaction to detect antibody labeling and hematoxylin counterstaining. Human tonsil FFPE tissues with and without primary antibody were used as positive and negative controls, respectively, with each run of IHC staining. IHC in tissue samples were evaluated by light microscopy (Axioscope; Carl Zeiss Microscopy, Jena, Germany) and reviewed by two NET expert pathologists who were blinded to all clinical data. In our study in most cases only bioptic specimen were available (40/68 specimen) which have been analyzed in total. In larger tumor samples, representative tumor sections were cut and immunostained; the tumor tissue of the whole slides was included into the analysis. In addition, for PD-L1 expression, special emphasis was placed to the tumor margins (whenever present) and to hot spots with high proliferative activity, given the fact that some previous publications describe increased expression in those areas. We interpreted results based on published criteria that have been well established for Non-Small-cell-Lung-Cancer (NSCLC) adopting the tumor proportion score (TPS) system (43–45). In accordance with these previously described criteria, only complete circumferential or partial cell membrane membranous PD-L1 expression positivity of viable tumor cells was scored, whereas cytoplasmatic PD-L1 staining in tumor cells was disregarded. The TPS was quantified by evaluating the ratio of PD-L1 positive tumor cells to the number of all viable tumor cells. Based on PD-L1 positive cell proportion, four categories were distinguished: 0 (no staining, < 1%), 1 (weak staining, 1%), 2 (moderate staining 1–49%), or 3 (strong staining, ≥49%). When computing TPS, the whole tumor area comprised within the tumor sample was considered, either for small biopsies as for samples embedded in total. Due to the high proliferative activity of NEN G3 the commonly used minimum of ≥100 viable carcinoma cells was easily fulfilled in all analyzed cases. Necrotic areas were excluded from scoring. PD-L1 expression on TCs and on ICs was scored once separately and once together. The density of cells expressing CD3, CD8, and CD68 was evaluated using a progressive classification: 0 (no staining), 1+ (weak staining), 2+ (moderate staining), or 3+ (strong staining) and considering only the intratumoral compartment. For this analysis, each sample examined was overlapped with the HE slide and with sequential IHC slides to evaluate each marker at the same location of the tumor and to cover the whole surface of the tumor comprised in the sample. Representative stains of the investigated tissues are shown in Figure 1. Sample images were obtained using Nano Zoomer 2.0-HT C9600 series and NDP.view 2.5.19 (both by Hamamatsu Photonics, Hamamatsu City, Japan).

### Tumor Microenvironment Classification

Finally, we characterized the four types of tumor microenvironment described by Teng and colleagues (20). For this analysis, we combined overall PD-L1 expression in the intratumoral compartment ≥1 with T cells (CD3+ score ≥1), as proposed by previous studies (20, 46).

### Statistical Methods

The Chi-Square or Fisher exact tests were used to examine differences in categorical variables, whereas the Wilcoxon rank-sum test and Mann-Whitney test were used to detect differences in continuous variables between groups of patients. Survival analysis could not be performed due to the lack of OS data. The statistical software program SPSS version 23 (SPSS, Inc., Chicago, IL, USA) was used to perform the computations for all analyses.

## Results

### Patient Characteristics

Fifty-seven patients with NEN G3 were enrolled in this study, from whom additional clinical information was available for 37 patients (64.9%). Thirty-four of the enrolled patients (59.6%) were male, median age at diagnosis was 67 years (range, 41–87 years) (Table 1). Tumors were mainly localized in the GEP tract (21 patients [36.8%]) and the lung (16 patients [28.1%]). 20 (35%) NEN originated from other locations: 8 (14%) from the genitourinary system, 4 (7%) from the ear, nose and throat (ENT) mucosa, 1 (1.8%) NEN was a Merkel cell carcinoma and 7 (12.3%) were considered cancers of unknown primary (CUP) (Table 1).

**Table 1:** Clinicopathological data and PD-L1 expression overall; PD-L1 positive cases on tumor cells and on immune cells.

**Category**		**PD-L1 positive**	**PD-L1 negative**	**IC PD-L1 positive**	**TC PD-L1 positive**	**Tot**
Gender, *n* (%)	Female	10 (43.5%)	13 (56.5%)	8 (34.8%)	5 (21.7%)	23
	Male	8 (23.5%)	26 (76.5%)	6 (17.6%)	4 (11.8%)	34
Mean age at diagnosis (range)		67 (41-87)	66 (44-79)	67 (41-87)	67 (43-81)	57
Primary tumor, *n* (%)	GEP	6 (28.6%)	15 (71.4%)	3 (14.3%)	3 (14.3%)	21
	Lung	4 (25%)	12 (75%)	4 (25%)	2 (12.5%)	16
	Other	8 (40%)	12(60%)	7(35%)	4(20%)	20
	Genitourinary tract	3 (37.5%)	5 (62.5%)	3 (37.5%)	0	8
	ENT	2 (50%)	2 (50%)	2 (50%)	2 (50%)	4
	MCC	1 (100%)	0 (0%)	1 (100%)	0 (0%)	1
	CUP	2 (28.6%)	5 (71.4%)	1 (14.3%)	2 (28.6%)	7
Disease extension, *n* (%)	Locally confined	2 (25%)	6 (75%)	2 (25%)	1 (12.5%)	8
	Locally advanced	9 (56.3%)	7 (43.8%)	9 (56.3%)	3 (18.8%)	16
	Extended	7 (24.1%)	22 (75.9%)	3 (10.3%)	5 (17.2%)	29
	Missing data		4			
Histology, *n* (%)	NET	0	3 (100%)	0	0	3
	NEC	17 (35.4%)	31 (64.6%)	14 (29.2%)	8 (16.7%)	48
	MiNEN	1 (16.7%)	5 (83.3%)	0	1 (16.7%)	6
Mean Ki-67 index (range)		80 (40-90)	70 (21-100)	80 (40-90)	80 (40-90)	52

*CUP, cancer of unknown primary; ENT, ear-nose-throat; GEP, gastro-entero-pancreatic; IC, immune cells; MCC, Merkel cell carcinoma; MiNEN, mixed neuroendocrine-nonneuroendocrine neoplasm; NEC poorly differentiated neuroendocrine carcinoma; NET, well-differentiated neuroendocrine tumor; PD-L1, programmed cell death ligand 1; TC, tumor cells; Tot, total*.

The disease was extended in most of the patients (49.1%), locally confined in 8 (14%) and locally advanced in 17 (29.8%) patients. Histological examination identified 3 (5.3%) patients with well-differentiated G3 lesions (NET G3), 48 (84.2%) patients with poorly differentiated NECs and 6 (10.5%) patients with MiNENs. Proliferative activity was high with a median Ki-67 of 80% (range, 21–100%).

### PD-L1 Expression in NEN G3

PD-L1 expression was detected in 18/57 (31.6%) of NEN G3. PD-L1 was more frequently expressed on IC than on TC (24.5 vs. 15.7%). PD-L1 positive tumor-infiltrating IC included myeloid cells, i.e., macrophages and dendritic cells, and lymphocytes. PD-L1 staining predominantly was weakly positive with a score of 1+ in almost all PD-L1 positive cases (16/57; 28%) on ICs and/or on TCs. As exceptions 1 sample (1.8%) showed strong (3+) PD-L1 expression on TC and 1 sample showed moderate (2+) PD-L1 expression on IC.

**Figure 2:**
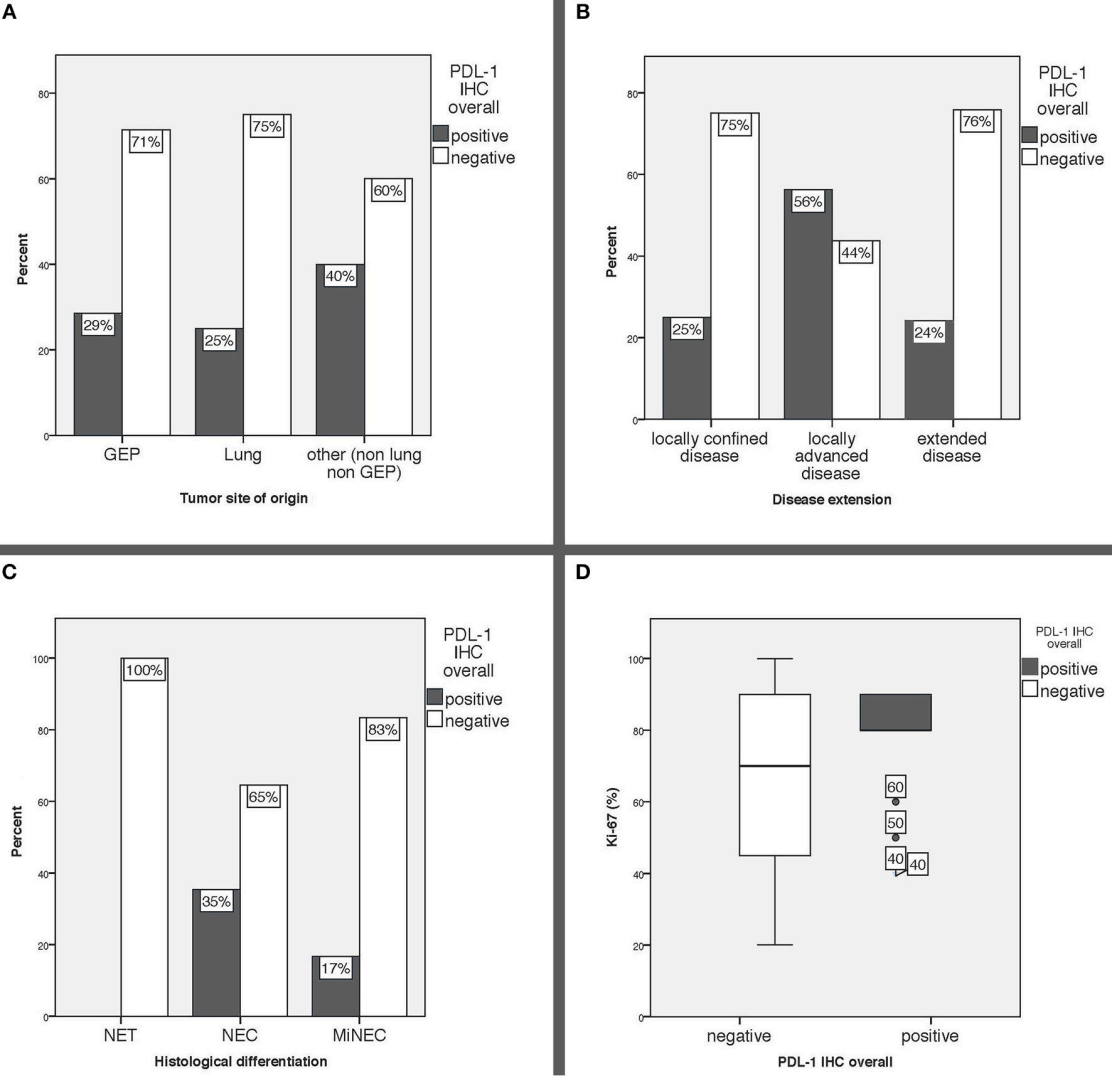
**(A)** PD-L1 expression overall in relation to tumor site of origin, **(B)** PD-L1 expression overall in relation to disease extension, **(C)** PD-L1 expression overall in relation to histological differentiation, **(D)** Ki-67 index expressed in % according to PD-L1 status. GEP: gastro-entero-pancreatic, other: includes 8 tumors originated from the genitourinary system, 4 from the ear, nose and throat mucosa, 1 Merkel cell carcinoma and 7 cancers of unknown primary, IHC: immunohistochemistry.

PD-L1 expression was comparable in GEP and lung NEN and tended to be higher in NENs originating from other sites of origin (GEP-NENs 28.6%; lung-NENs 25%; other NENs 40%) (Table 1). When evaluating PD-L1 expression with respect to histological differentiation, none (0/3) of the well-differentiated NET was positive, whereas 35.4% of NEC and 16.7% of MiNEN were PD-L1 positive. Moreover, intensity of PD-L1 expression was scored weak to moderate (range, 1–2) in all NECs, whereas the only PD-L1 positive MiNEN was scored strongly positive (3+). Median Ki-67 index was slightly higher in PD-L1 positive than in PD-L1 negative cases (80 vs. 70%). PD-L1 positivity was more common in patients who suffered from locally extended disease at diagnosis (56.3%) compared to those with locally confined or extended disease (25 and 24.1%, respectively). PD-L1 expression in relation to major clinical characteristics is reported in Figure 2.

In 11 patients 2 different tumor samples were available. In these patients, PD-L1 expression was highly discordant with 3 patients having a positive result in only 1 of the 2 samples. Moreover, in the only patient who expressed PD-L1 in both samples PD-L1 was detected on both cell types, TC and IC, in one sample but only on TC on the other one.

When analyzing the association of PD-L1 expression either on TC and/or IC with respect to patient characteristics such as gender and age at diagnosis, NEN sites of origin, disease extension, histological differentiation, and Ki-67 index no association was found. The clinical and histopathological characteristics of each patient expressing PD-L1 as well as the distribution of PD-L1 positive cases with regard to clinicopathological data are reported in Supplementary Table 1.

### TAIC Analysis and T Cell/PD-L1 Histological Patterns

TAIC analysis was possible to explore in 22 cases. Infiltration of the tumors by T cells was more frequently observed than infiltration by cytotoxic T cells. CD3+ T cells and CD68+ tumor-infiltrating macrophages/dendritic cells were detected in about half of the samples (45.5 and 59.1%, respectively), whereas CD8+ cytotoxic T cells were rarely present in the tumors (18.2%). Interestingly we could not detect any CD8+ margin of invasion as described in other studies concerning non-NEN cancer: if present CD8+ cells where homogeneously distributed within the tumor areas. Consequently, we did not observed any PD-L1 hotspot in relation to CD8+ infiltration as previously described in other cohorts (47). Macrophage/dendritic cell infiltration was detected even in the absence of intratumoral T cells. Moreover, the density of T cells and cytotoxic T cells within the tumor was low in all cases (score ≤ 1), whereas intratumoral macrophages/dendritic cell infiltration was more intense (range, 0–3).

**Figure 3:**
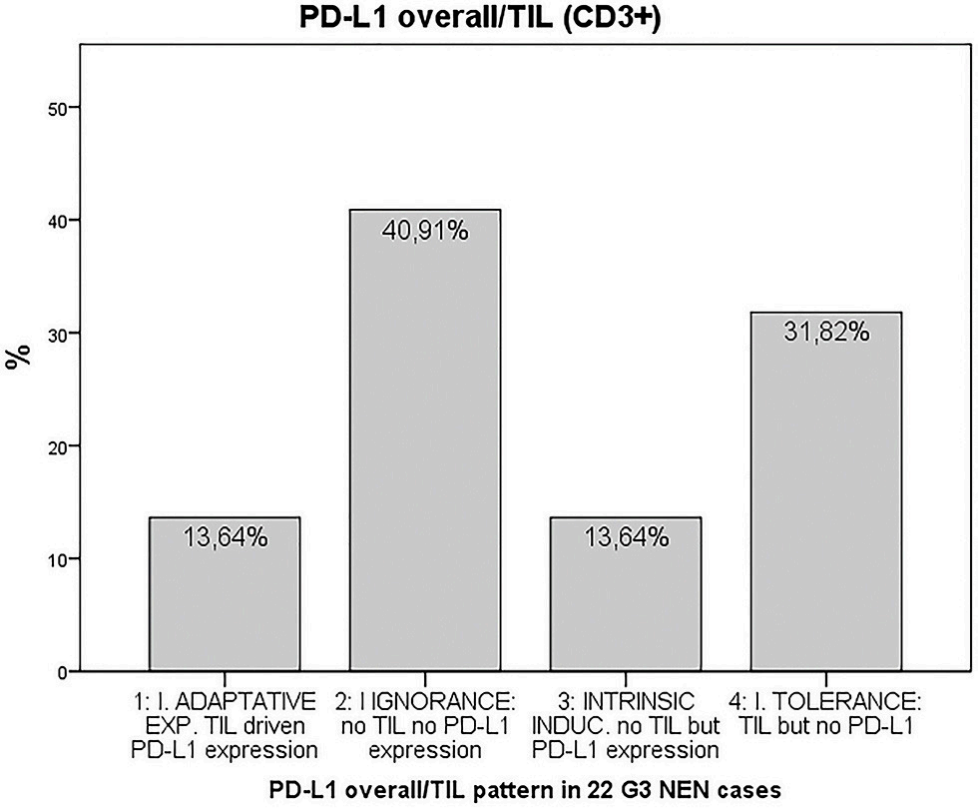
Tumor microenvironment classification according to the PD-L1 overall/TIL (CD3+) pattern (20) in 22 G3 NEN cases. PD-L1: programmed cell death protein ligand 1, TIL: tumor-infiltrating lymphocytes.

Finally, we provided evidence for the presence of tumor microenvironment patterns in NEN G3, based on PD-L1 expression and T cell infiltration, as described by Teng et al. (20). In total, 72.7% of the 22 tumor samples included in the final analysis were PD-L1 negative (Figure 3). 40.9% of the 22 samples were PD-L1 negative but lacked T cell infiltration, 31.8% were PD-L1 negative and showed T cell infiltrates. 27.2% of the NEN samples were PD-L1 positive, half of which were without and half with T cell infiltration. Details of PD-L1 expression and T cell infiltration for each tumor sample are shown in Supplementary Table 2.

## Discussion

We demonstrate that 18/57 (31.6%) of neuroendocrine G3 lesions express PD-L1 and that its expression was uniformly of low intensity. Within the intratumoral compartment, we found that PD-L1 expression was more frequent on IC than on TC, thus confirming previous reports (33, 34, 36, 41, 48). The proportion of GEP (28.6%) and lung (25%) NEN expressing PD-L1 was similar, slightly more PD-L1 is expressed by NEN of other locations (40%).

Our data on the frequency of PD-L1 positive GEP-NEN G3 is consistent with that reported by Roberts *et al*. in 37 GEP NEC (32%) (41) and by Kim et al. in 17 pancreatic NEN G3 patients (41%) (38). However, in another small series, PD-L1 expression was found in all of 9 NEN G3 patients (40). Previous results on PD-L1 expression in lung NEN vary considerably: Fan et al. reported PD-L1 positivity in as much as 58.8% of 80 lung NEN patients (37), and Tsuruoka et al. (*n* = 227) detected PD-L1 expressed in 10.4% of large cell and 5.8% of small cell NEC (42). Recently, Kasajima et al. reported PD-L1 immunoreactivity in IC of 73 (39%) lung NEC, while TC were labeled in only 21 (11%) cases (48), which is in line with our own results. PD-L1 expression in NEN from other sites of origin was described in up to 55% MCC (33) and in 5/8 (62.5%) head and neck NENs (39), similar to our results. To our knowledge, no PD-L1 expression data has been reported in NEN of other sites of origin.

In our study PD-L1 was most frequently expressed in NECs and to a lesser extent in MiNENs, but not in NET G3. Furthermore, more than half of the patients who presented with locally extended disease at diagnoses showed PD-L1 immunoreactivity, compared with only a quarter of the patients with locally confined or extended disease. Although only a limited number of NET G3 were included in our study, our data therefore confirm others who demonstrated PD-L1 expression mainly on TC of poorly rather than of well-differentiated NENs (49, 50) or who even exclusively detected PD-L1 expression in GEP-NEC (51). These results indicate, that the more aggressive the tumor, the higher the expression of PD-L1. Taking into account that NEC also are more frequently associated with high-level microsatellite instability and high mutational load than well-differentiated NEN (52–54), poorly differentiated NEN might represent a preferred target for immunotherapy. In other PD-L1 studies based on non-NEN patients, the tumor margin gained attention as a preferential site of CD8+ invasion associated with PD-L1 hotspot expression in the invasive margin has been observed. Therefore, the tumor margin was systematically analyzed in all our samples when visible. However, we did not detect any CD8+ margin of invasion: when present, CD8+ cells where homogeneously distributed within the tumor areas and consequently we did not observed any PD-L1 hotspot in relation to CD8+ infiltration. Analysis of multiple samples showed high intrapatient variability of PD-L1 expression, suggesting that its expression might change over time, with tumor treatment or even evolve during disease progression. However, further studies are necessary to better understand reproducibility of PD-L1 assessment, intra-patient and intra-lesional heterogeneity, and protein expression dynamic changes over time.

Recent evidence has pointed out that in terms of therapeutic implications PD-L1 expression should be best interpreted in the context of intratumoral T cell infiltration (23). We observed infiltration of the tumor by macrophages/dendritic cells as well as by T cells in almost a half of the cases but cytotoxic T cells in only few cases. T cells and cytotoxic T cell density within the intratumoral compartment was low. PD-L1 expression depends on many factors including methodological issues such as the type of antibody as well as tumor microenvironment and tumor treatment (55, 56). PD-L1 expression therefore is a highly dynamic immunological parameter of anticancer immunity status rather than a predictive parameter. Hence, lack of detectable PD-L1 expression does not preclude antitumor activity in response to anti-PD-1/PD-L1 treatment. Accordingly, there is a need for more accurate biomarkers for response to immune checkpoint inhibitor treatment.

The characterization of the tumor microenvironment patterns according to the classification by Teng and colleagues (20) showed that more than 40% of the tumors had an immune ignorant phenotype, defined as no PD-L1 expression and absence of TILs. In these tumors single agent checkpoint blockade probably would not be successful and combination treatment to enhance T cell infiltration into the tumors and then avoid them being turned off would have to be considered (20). About 14% of the specimens expressed PD-L1 in the presence of TILs. These patients most likely would benefit from single agent anti-PD-1/L1 blockade.

Given the rareness of neuroendocrine G3 neoplasia and the strong clinical need for novel therapeutic options in these very aggressive tumors with a dismal prognosis, our study adds valuable clinical information for the generation of future prospective studies and helps in developing new therapeutic strategies in these patients. Since there are no established and clearly effective second line chemotherapy regimens in NEN G3, an immunotherapeutic approach seems to be a promising therapeutic strategy in these tumors which is currently followed in a number of clinical prospective trials including our own ongoing study with Avelumab in patients with NEN G3 and documented tumor progression after first-line chemotherapy (AveNEC ClinicalTrials.gov Identifier: NCT03352934). In these studies, preliminary data indicate that only a minority of patients with NEN G3 shows a significant and clinically highly relevant response to a monotherapy with a single immunotherapeutic agent. This is in accordance with the data of the present study, which indicate that the majority of these tumors exhibits only a low amount of PDL1 expression and tumor infiltrating immune cells thus suggesting that a dual checkpoint inhibition or combination therapies with additional radiation or chemotherapy which could increase the immunogenicity of these tumors could be a promising strategy. In the present study investigated PD-L1 expression in a large cohort of NEN G3 of different sites of origin in the context of the tumor immune microenvironment. Taken together checkpoint inhibitor therapy is likely to be effective in a subgroup of NEC patients and this needs to be defined further. However, validation of our findings in a larger cohort of patients with bigger tumor specimens available for IHC analyses and more detailed clinical information is needed. Additionally there is still a significant heterogeneity among the available tests for PD-L1 analysis, with different antibodies used and a missing uniform and standardized definition of the threshold for PD-L1 positivity. In addition, these significant methodological variations are also in part reflected by yet unclear clinical and prognostic consequences which also vary according to tumor type and the time point of the tumor sample collection with regard to tumor progression and current therapy of the patient (57–61).

Future research has to take into account the potential of synergistic combinations with immune checkpoint inhibitors (62). Apart from targeting different or other checkpoints than CTLA-4 or PD-1/PD-L1 combinations with classic and newer cancer therapies including radiotherapy, chemotherapy and targeted therapy as well as other immunotherapies such as tumor vaccines and adoptive cell therapy are going to attract interest.

## Ethics Statement

This study was approved by the local ethical committee [Ethik-Komission der Landestekammer Rheinland-Pfalz, Nr.: 837.120.15 (9888)] with written informed consent from all subjects.

## Author Contributions

MW and CF share senior authorship. MW, CF, WR, and MF designed the study. TM, KB, JK, SB, ES, and WR were involved in planning and supervising the work. AS, SZ, and MF performed the experiments and analyzed the data. MF wrote the paper with input from all authors.

### Conflict of Interest Statement

MW and CF received honoraria as a speaker and board member as well as research funding from Novartis, Ipsen, and Merck. The remaining authors declare that the research was conducted in the absence of any commercial or financial relationships that could be construed as a potential conflict of interest.
