# Supplementary material for: PD-L1 Expression and Immune Cell Infiltration in Gastroenteropancreatic (GEP) and Non-GEP Neuroendocrine Neoplasms With High Proliferative Activity
Source: Front Oncol. 2019 May 7;9:343. doi: 10.3389/fonc.2019.00343 (PMC6514221; doi:10.3389/fonc.2019.00343)
Supplement: Supplementary file 1 [file Data_Sheet_1.docx]

Supplementary Material

**PD-L1 expression and immune cell infiltration in gastroenteropancreatic (GEP) and non-GEP neuroendocrine neoplasms with high proliferative activity**

**Authors**

Martina Ferrata, Arno Schad, Stefanie Zimmer, Thomas J. Musholt, Katharina Bahr, Julian Kuenzel, Sven Becker, Erik Springer, Wilfried Roth, Matthias M. Weber* & Christian Fottner

*** Correspondence:** Prof. Dr. Matthias M. Weber: matthias.weber@unimedizin-mainz.de

**Supplementary Tab. 1. Histopathological and clinical characteristics of the PD-L1 positive cases**

| **Sample** | | | | | **PD-L1 expression** | | **NEN tumor characteristics** | | | **Routine IHC** | | | **Patients characteristics** | |
| --- | --- | --- | --- | --- | --- | --- | --- | --- | --- | --- | --- | --- | --- | --- |
| ID | No.^a^ | Type | Dimension | Site | TC^b^ | IC^b^ | Histologic Type | Site of origin | Extension^c^ | Ki-67 (%) | CgA | Syn | Gender | Age^d^ |
| 7 | 1 | b^4^ | Small | MET | 1 | 0 | NEC | Pancreas | III | 90 | 1+ | 2+ | M | 59 |
| 13 | 1 | b | Medium | MET | 0 | 1 | NEC | Lung | II | 80 | 1+ | 2+ | F | 67 |
| 14 | 1 | tot^5^ | Large | P | 0 | 1 | NEC | Urinary system | I | 50 | 2+ | 1+ | F | 87 |
| 16 | 2 | b | Large | MET | 0 | 1 | NEC | Lung | III | 80 | 1+ | 1+ | M | 44 |
| 19 | 1 | b | Large | P | 0 | 1 | NEC | Prostate | III | 90 | 2+ | 2+ | M | 72 |
| 24 | 1 | b | Small | M | 1 | 0 | NEC | CUP | III | 80 | 2+ | 1+ | M | 81 |
| 33 | 1 | b | Large | P | 0 | 1 | NEC | Urinary system | II |  | 3+ | 3+ | F | 80 |
| 37 | 1 | tot | Large | N^9^ | 0 | 1 | NEC | MCC | II | 90 | 1+ | 3+ | M | 55 |
| 38 | 2 | tot | Large | P | 0 | 1 | NEC | Colon | II | 80 | - | 2+ | M | 82 |
| 39 | 1 | b | Small | MET | 1 | 0 | NEC | Gastrinoma | III | 40 | 3+ | 2+ | F | 50 |
| 40 | 1 | tot | Large | P | 1 | 1 | NEC | Lung | II | 90 | 2+ | 1+ | F | 68 |
| 41 | 1 | b | Small | MET | 3 | 0 | MiNEN | Colon | III | 60 | 2+ | 2+ | F | 55 |
| 45 | 2 | tot | Large | P | 0 | 1 | NEC | Colon | II | 40 | - | - | F | 41 |
| 50 | 1 | tot | Large | P | 0 | 1 | NEC | Pancreas | II | 90 | 1+ | 2+ | F | 72 |
| 51 | 1 | tot | Large | N | 1 | 2 | NEC | ENT | II | 90 | 2+ | 2+ | F | 67 |
| 52 | 1 | tot | Large | N | 1 | 2 | NEC | Lung | II | 80 | 2+ | 2+ | M | 75 |
| 54 | 2 | tot | Medium | N | 1 | 1 | NEC | CUP | II | 80 | 1+ | 1+ | F | 43 |
| 56 | 1 | b | Small | P | 1 | 1 | NEC | ENT |  | 90 | 2+ | 2+ | M | 67 |
| Abbreviations: b: biopsy sample, CgA: chromogranin A, CUP: cancer of unknown primary, ENT mucosa: mucosa of the ear-nose-throat tract, F: female, IC: immune cells, ID: identification number, IHC: immunohistochemistry, M: male, MCC: Merkel cell carcinoma, MET: distant metastases, MiNEN: mixed neuroendocrine-nonneuroendocrine neoplasm, N: lymph node, NEC: neuroendocrine carcinoma, NEN: neuroendocrine neoplasm, PD-L1: programmed cell death protein ligand 1, P: primary tumor, Syn: synaptophysin, TC: tumor cells, tot: lesion embedded “in toto”  a. number of samples  b. D-L1 expression: 0 (no staining, <1%), 1 (weak staining, 1%), 2 (moderate staining 1–49%), or 3 (strong staining, ≥49%)  c. defined as: I - locally confined, II - locally advanced, III - extended disease  d. at diagnosis | | | | | | | | | | | | | | |

**Supplementary Tab. 2: PD-L1 status and tumor-associated immune cell density**

| **ID** | **Primary tumor** | **Histology** | **Ki-67 (%)** | **PD-L1 on IC** | **PD-L1 on TC** | **PD-L1 overall** | **CD3** | **CD8** | **CD68** | **TIL/PD-L1 pattern*** |
| --- | --- | --- | --- | --- | --- | --- | --- | --- | --- | --- |
| 14 | Genital tr. | NEC | 50 | 1 | 0 | 1 | 1 | 0 | **0** | Type 1: adaptive immune resistance |
| 40 | Lung | NEC | 90 | 1 | 1 | 1 | 1 | 0 | **2** | Type 1: adaptive immune resistance |
| 56 | ENT | NEC | 90 | 1 | 1 | 1 | 2 | 2 | **3** | Type 1: adaptive immune resistance |
| 4 | Rectum | NEC | 90 | 0 | 0 | 0 | 0 | 0 | **0** | Type 2: immune ignorance |
| 8 | Lung | NEC | 90 | 0 | 0 | 0 | 0 | 0 | **1** | Type 2: immune ignorance |
| 11 | Lung | NEC | 80 | 0 | 0 | 0 | 0 | 0 | **1** | Type 2: immune ignorance |
| 12 | Rectum | MiNEN | 90 | 0 | 0 | 0 | 0 | 0 | **0** | Type 2: immune ignorance |
| 20 | Colon | NEC | 70 | 0 | 0 | 0 | 0 | 0 | **1** | Type 2: immune ignorance |
| 21 | Pancreas | NET | 30 | 0 | 0 | 0 | 0 | 0 | **1** | Type 2: immune ignorance |
| 22 | Gallbladder | MiNEN | 70 | 0 | 0 | 0 | 0 | 0 | **0** | Type 2: immune ignorance |
| 36 | Colon | NEC | 70 | 0 | 0 | 0 | 0 | 0 | **0** | Type 2: immune ignorance |
| 44 | Lung | NEC | 50 | 0 | 0 | 0 | 0 | 0 | **0** | Type 2: immune ignorance |
| 38 | Colon | NEC | 80 | 1 | 0 | 1 | 0 | 0 | **1** | Type 3: constitutive expression |
| 45 | Colon | NEC | NA | 1 | 0 | 1 | 0 | 0 | **3** | Type 3: constitutive expression |
| 50 | Pancreas | NEC | 90 | 1 | 0 | 1 | 0 | 0 | **0** | Type 3: constitutive expression |
| 2 | Genital tr. | NEC | NA | 0 | 0 | 0 | 1 | 0 | **2** | Type 4: immune tolerance/INF γ res. |
| 15 | Stomach | NET | 25 | 0 | 0 | 0 | 1 | 1 | **0** | Type 4: immune tolerance/IFN γ res. |
| 17 | Lung | NEC | 90 | 0 | 0 | 0 | 1 | 0 | **1** | Type 4: immune tolerance/IFN γ res. |
| 34 | CUP | NET | 30 | 0 | 0 | 0 | 1 | 0 | **2** | Type 4: immune tolerance/IFN γ res. |
| 47 | Colon | MiNEN | 40 | 0 | 0 | 0 | 1 | 0 | **0** | Type 4: immune tolerance/IFN γ res. |
| 49 | CUP | NEC | 30 | 0 | 0 | 0 | 1 | 1 | **2** | Type 4: immune tolerance/IFN γ res. |
| 57 | ENT m. | NEC | 75 | 0 | 0 | 0 | 1 | 1 | **2** | Type 4: immune tolerance/IFN γ res. |
| CUP: cancer of unknown primary; ENT: ear-nose-throat mucosa; IC: immune cells; ID: identification number; IFN: interferon, MiNEN: mixed neuroendocrine-nonneuroendocrine neoplasm; NEC poorly differentiated neuroendocrine carcinoma; NET: well-differentiated neuroendocrine tumor; PD-L1: programmed cell death ligand 1; res.: resistance, TC: tumor cells; TIL: tumor infiltrating lymphocytes, tr.: tract  * according to the classification of Teng et al. (Teng *et al.* 2015) | | | | | | | | | | |
